# Supplementary material for: A qualitative investigation of dental internships in Saudi Arabia: Exploring the experiences of dental interns
Source: PLoS One. 2024 Jul 15;19(7):e0307310. doi: 10.1371/journal.pone.0307310 (PMC11249209; doi:10.1371/journal.pone.0307310)
Supplement: Attachment — Submitted filename: Response to reveiwers.docx [file pone.0307310.s001.docx]

*Responses to reviewers*

**Table 1.**

| *Commentor* | *Comment* | *Our response* |
| --- | --- | --- |
| Reviewer 1 | Reviewer #1: Abstract: The presented section is not balanced. They present little information about methodology, and many conclusions not directly related to the objective. Authors should rebalance their abstracts | Dear reviewer 1,  Thank you for your feedback.  To address your comment, the abstract has been rebalanced, as you suggested, by making several changes:   1- Clarifying and accentuating the knowledge gap. 2- Adding more details to the method section. 3- Relating the conclusion to both the results and the objective of the study.   Please see line 26-54 |
| Reviewer 1 | Background: The authors could be more objective in this section. A lot of information is presented that does not help the reader in understanding the problem or aim studied. Affirmation presented in lines 135-137 need references; The hypothesis or assumption investigated are not clear. | Thank you for your comment.  To address your comment, major changes have been made to the introduction section to enhance objectivity and to clarify the study's problem and aim. We have removed several sentences and references, while introducing new information to improve the flow of ideas.  The revised background now begins by introduction about dental internship year, including its various names across different countries, and highlighting its importance and objectives. We then proceeded to describe the primary challenges faced by interns. Additionally, we have outlined the factors that contribute to the success of dental interns during this year. Finally, we have included a paragraph emphasizing that previous studies have predominantly focused on the clinical aspects of the internship experience, overlooking its other aspects and the overall experiences. This paragraph can be very helpful to introduce the reader to the study's problem and aim.  We would like to kindly note that in qualitative research there is no hypothesis, but authors should mention the aim of their study.  Please see line 56-111 |
| Reviewer 1 | Methodology: Research undersection could receive part of background. If the population of study is known (dental interns who had earned bachelor’s degrees in dentistry from two universities in Saudi Arabia), why was snowball technique applied? | Thank you for your comment.  Snowball sampling involves asking the initially recruited participants to help us to find additional participants who also meet the criteria. This method is helpful to recruit relevant participants.  In our research, we used this approach by recruiting dental interns. However, since we were unable to approach all of them directly, we asked the first participants to guide us in finding others who fit the criteria. We stated this clearly in the method section.  Please see line 142-145 |
| Reviewer 1 | As presented in 230 and 231, a qualitative study demands a “literature review or existing theory” to interpret data processed with thematic analysis. However, authors were not clear in the method or background sections. I encourage authors to create an undersection with literature indicators or framework theory used to interpret qualitative data. | We appreciate your feedback.  However, it seems there may have been a misunderstanding.  We actually took a different approach in our research. *We did not use any preexisting theory or specific framework for data analysis to reveal it.* Thus, our approach in data analysis is classified as *inductive* rather than *deductive.* We made this explicitly clear in our method section (data analysis subsection), stating, *“Inductive thematic analysis was applied in this research whereby the themes were taken from the data collected and were not prepared based on a literature review or review of existing theory.”*  By generating the themes primarily from the dental interns' experiences, we believe this strengthens the study, as the data generated are thus grounded in the real-life experiences of the participants, rather than being influenced by any imposed theoretical framework or preexisting theory.  Please see line 220-22 |
| Reviewer 1 | There is nor a statement locating the researcher culturally or theoretically. Influence of the researcher on the research, and vice- versa, was not addressed. | Special thanks for this comment and the subsequent one.  We agree that discussing the influence of the researcher's theoretical background on the research process (known as reflexivity) and acknowledging the philosophical position of the researcher is important and improves the overall presentation of the paper.  We have not mentioned these details in the first version of the manuscript because we rarely see papers discussing them. However, we recognize this as a valuable opportunity to address them.  Thus, to address your comment, we added a subsection titled "Researcher Characteristics and Reflexivity" under the method section. In this subsection, we acknowledged what we believe to be potential impacts on the research process: 1) the researcher's previous experience and 2) their theoretical background.  However, we would like to kindly note that, in this particular study, we don't feel that cultural background may significantly influence the research process unless it occurs completely subconsciously. Therefore, in this subsection, we described what we believed to be a potential influence.  Please see line 237-251 |
| Reviewer 1 | The methodology section could benefit it authors clarify the philosophical perspective that guide the study  After this, reviewers would answer the following question: “Is there congruity between the stated philosophical perspective and the research methodology?” | Thank you for your comment.  To address it, we have added a subsection under the method section titled "Philosophical Perspective." In this subsection, we describe our philosophical perspective, including our ontological position, and explain how it informs our epistemological stance and methodology. By doing so, we demonstrate the alignment between our philosophical perspective and the chosen methodology.  Please see line 113-129 |
| Reviewer 1 | Results are well presented, including students’ speeches. However, the authors did not present the characterization of students included in the study. | Thank you for your feedback.  There is a subsection in the Method section titled "Research Subjects/Participants." Under this subsection, we present some characteristics of the study participants, such as gender, age, and the number of months they have completed the internship. However, to address your comment, we have added additional details about the study participants, aiming to benefit the reader and enhance the value of the manuscript.  Also, we would like to kindly note that we understand that you provided this comment in the Results section, but we prefer to address it in the method section as based on the recommendations outlined in the O'Brien key reference paper titled "Standard for Reporting Qualitative Research: Synthesis of Recommendations," which we have cited, it would be more appropriate to address this comment in the Method section. This ensures that our paper adheres to the recommended reporting standards in the field of qualitative research. Thank you again for your comment.  Please see line 156-161  ***Finally, we hope this version of the manuscript meets your expectations.*** |
| Reviewer 2 | To improve knowledge in this field, it would be important to include negative feelings regarding internship. Considering only the positive aspects offers a superficial view of the topic. It would also be important to analyze the role of supervisors/tutors in students' opinions | Dear reviewer 2,  Thank you for your feedback.  We fully agree with your viewpoint that including the negative aspects of the internship would undoubtedly enhance the knowledge in this area. Additionally, including perspectives of supervisors and other relevant individuals (for example, program directors) would certainly contribute to gaining a complete understanding of the internship and dental interns’ experiences.  However, we would kindly note that examining these areas properly would require several separate studies and research questions.  However, to address your comments, we have added these points as limitations of our study by acknowledging the aspects we did not cover and utilizing these points as suggestions for future research directions.  Please see line 623-627  Please see line 637-642 |
| Reviewer 2 | Another aspect to be taken into account is whether there is any influence of gender on the responses. | Thank you for your comments.  It's very interesting to consider if there are differences in experiences between males and females during internships.  However, we would like to kindly note that our chosen *qualitative methodology* does not enable us to specifically analyze the differences between males and females.  Qualitative research is employed to generates patterns from data rather than detect statistical differences between, for example, males and females.  To address your comment, we have acknowledged this point as one of the limitations of our study. Also, we have used it as a suggestion for future research directions.  Please see line 627-632  Please see line 642-645  ***Finally, we hope this version of the manuscript meets your expectations.*** |
