# Supplementary material for: A qualitative investigation of dental internships in Saudi Arabia: Exploring the experiences of dental interns
Source: PLoS One. 2024 Jul 15;19(7):e0307310. doi: 10.1371/journal.pone.0307310 (PMC11249209; doi:10.1371/journal.pone.0307310)
Supplement: Attachment — Submitted filename: Response to Reveiwers.docx [file pone.0307310.s003.docx]

**Response to reviewers**

| Commentor | Comment | Response to reviewers |
| --- | --- | --- |
| Journal Requirements | “Please review your reference list to ensure that it is complete and correct. If you have cited papers that have been retracted, please include the rationale for doing so in the manuscript text, or remove these references and replace them with relevant current references. Any changes to the reference list should be mentioned in the rebuttal letter that accompanies your revised manuscript. If you need to cite a retracted article, indicate the article’s retracted status in the References list and also include a citation and full reference for the retraction notice.” | Dear Reviewer,  Thank you for your feedback.  In response to your comments, we would like to kindly let you know that we have reviewed the references again to ensure their correctness and consistency. Additionally, we have not cited a retracted paper.  Furthermore, during the first round of revision, some changes were made to the reference list. One of the reviewer's comments highlighted that our background section was unclear and contained irrelevant information. Consequently, we carefully reviewed the background section again and removed the irrelevant information. As a result, some references were also removed accordingly since they were no longer cited.  For example, the references that were removed are as follows:   1- Alsoghier A, Ali K. Preparedness of newly qualified dentists in Riyadh, Saudi Arabia [internet]. Oct. Accessed July 31, 2023. Available from: https://www.researchsquare.com/article/rs-2104655/v1; in review; vol 2022. 2- Bahammam LA, Linjawi AI. Scholar outcomes for dental internship research program in Saudi Arabia: A qualitative evaluation. Med Teach 2017;39:S80-S85. 3- Prince K, Van De Wiel M, Van Der Vleuten C, Boshuizen H, Scherpbier A. Junior doctors' opinions about the transition from Medical School to clinical practice: A change of environment. Educ Health (Abingdon). 2004;17:323-331. 4- Lalloo R, Johnson NW, Blinkhorn AS, Ichim P. Australian dental students' views on a compulsory internship scheme. Health Educ J 2011;70:468-474.   In addition, one reference has been replaced. The reference "Riyadh Elm University. Internship training Program [internet]. Available from: https://www.riyadh.edu.sa/internship-training-program/" has been replaced with a reference to "King Khalid University. Dental Internship Program Committee [Cited 2023 August 704 07]. Available from: https://dent.kku.edu.sa/en/node/218." This change was made due to the expiration of the previous reference webpage.  ***However, all remaining cited information in the paper is now properly referenced in the reference list.***  Thank you again for your comment. We hope this version of the manuscript meets your expectations. |
| Reviewer 3 | “In the methods, it would be important for the authors to explain the coding used P1…P2..” | Dear reviewer,  Thank you for your comment. To address your comment, we have added a paragraph in the method section explaining the codes (P1, P2, P3…) used in the results section.  Please see line 255-257 |
| Reviewer 3 | “The conclusion is too long. It could be clearer and more direct” | Thank you for your feedback.  To address your comment, we have shortened the conclusion of this manuscript with the aim of making it clearer and more concise.  Please see line 645-666  Thank you again for your comments. We hope this version of the manuscript meets your expectations. |
